# mpMRI‐based MGMT methylation status prediction for glioblastoma through off‐the‐shelf deep features: A multi‐dataset feasibility study

**DOI:** 10.1002/acm2.70373

**Published:** 2025-11-23

**Authors:** Junhua Chen, Zhanghong Wang, Banghua Yang

**Affiliations:** ^1^ Department of Psychiatry and Psychology Mental Health Center Affiliated to Shanghai University School of Medicine Shanghai China; ^2^ School of Mechatronic Engineering and Automation Shanghai University Shanghai China; ^3^ Department of Anesthesiology Wenzhou Hospital of Integrated Traditional Chinese and Western Medicine Wenzhou Zhejiang China

**Keywords:** delta deep features, MGMT promoter, off‐the‐shelf deep features, radiogenomics for glioblastoma

## Abstract

**Objectives:**

O‐6‐methylguanine DNA methyltransferase (MGMT) promoter methylation status is a critical prognostic factor in glioblastoma. The aim of this study is to evaluate the feasibility of diagnosing MGMT status in a rapid, non‐invasive manner using multiparametric magnetic resonance imaging (mpMRI). The proposed method seeks to reduce reliance on stakeholders, thereby facilitating potential clinical applications in the future.

**Materials and methods:**

This study employed a Siamese neural network (SNN) as the backbone of the model to effectively leverage information from various mpMRI modalities. Off‐the‐shelf deep learning features extracted from pre‐trained networks was used to represent the information from mpMRI and adopted as the inputs of SNN. Delta deep features from T1 modality were integrated as additional branch of SNN to enhance model's performance. Finally, external validation was performed to increase the robustness of study. The proposed method was applied to one of the largest publicly available mpMRI datasets, comprising 585 participants, with an additional 81 samples used for external validation.

**Results:**

The proposed method achieved an average area under the curve (AUC) of 0.666 with a standard error of the mean (SEM) of 0.031, average precision of 0.591 (SEM 0.021), and average recall of 0.630 (SEM 0.064). In external validation, the method yielded an average AUC of 0.624 (SEM 0.022), precision of 0.674 (SEM 0.050), and recall of 0.810 (SEM 0.101).

**Conclusion:**

The results demonstrate that our method outperforms existing approaches on a single‐CPU platform. Ablation studies confirmed the effectiveness of incorporating delta T1 deep features, while external validation confirmed the method's reliability across different datasets.

## 1 INTRODUCTION

Glioblastoma, the most aggressive primary brain tumor, affects approximately 3.2 per 100 000 people annually in the United States, with a median age at diagnosis of 64 years and a slightly higher incidence in males.^1^ The prognosis remains poor, with a median survival of 15–18 months despite advances in treatment.^2^ The status of O‐6‐methylguanine DNA methyltransferase (MGMT) promoter methylation is a critical prognostic factor in glioblastoma, as its methylation is associated with better response to alkylating agents like temozolomide, improving the overall survival.^3^ Patients with methylated MGMT have significantly longer median survival compared to those with unmethylated MGMT, statistical analyses report a median overall survival (mOS) of 22 months for the methylated group versus 12 months for the unmethylated group.^4^ The facts above emphasizing the importance of its assessment in treatment planning, patient management.^5^

The MGMT promoter methylation status in glioblastoma is typically determined through molecular methods such as methylation‐specific polymerase chain reaction (MSPCR) or pyrosequencing, with the latter providing quantitative analysis^6^, ^7^ Although these methods offered more precise information and used as gold standard in clinical, mentioned laboratory examinations for MGMT status are invasive, expensive, and time‐consuming. For instance, there are hours even days to make sure the MGMT status by using MSPCR in clinical practices.^8^ Moreover, MGMT testing is generally performed as part of a broader molecular profiling panel that may analyze dozens of genes. As a result, patients often must wait several weeks to receive their complete diagnostic report. The purpose of this study is making a feasibility study for diagnosing MGMT status in quick and non‐invasive manner, the method should reduce the various stakeholders for potential clinical applications in the future.

Multiparametric magnetic resonance imaging (mpMRI) is crucial for the diagnosis and treatment planning of glioblastoma, as it provides detailed images that facilitate accurate identification of tumor boundaries, monitoring of disease progression, and guidance for surgical and therapeutic interventions^9^.^10^ Although highly valuable, mpMRI's limitations in glioblastoma diagnosis include potential false negatives in early detection, and difficulty in differentiating true progression from treatment‐related effects.^11^ Developing an MGMT status classification system for glioblastoma based on MRI would eliminate the need for additional examinations, offering significant advantages for both diagnosis and treatment planning, this study will focus on this problem.

Imaging‐based MGMT promoter status classification falls under the field of radiogenomics,^12^ which examines how an individual's genetic makeup influences their response to medical imaging techniques and the imaging characteristics of diseases. The prediction of MGMT status in glioblastoma using radiogenomics began with studies focusing on radiomics signatures^13^.^14^ However, these early studies relied on hand‐crafted features, making the model‐building process labor‐intensive and subjective. Additionally, the inherent nature of signature‐based methods introduces the potential for coincidental feature selection and dataset bias, which can significantly impact the reported high performance.^15^

In deep learning era, multiple methods were proposed to finish this radiogenomics analysis task, traditional CNN methods and target domain data fine‐tune based methods reported high performance. For instances, Mun et al.^16^ proposed a multi‐modal late fusion 3D classification network and then achieved top performance at 0.698 for MGMT status classification; during an international MGMT radiogenomics analysis competition ‐BraTS 21(RSNA‐MICCAI Brain Tumor Radiogenomic Classification competition)‐ which supported by Radiological Society of North America (RSNA), the winner (across 1556 teams) achieved an area under receiver operating characteristics Curve (AUC) at 0.622 for this task.^17^ However, a recently published paper concerned the available of deep learning method in predicting MGMT status due to the negative results were reported—the fine‐tuned models achieved average AUC at 0.631 in five‐fold cross‐validation.^18^

The purpose of this study is to develop a new and training‐efficient deep learning model for predicting MGMT status using multiparametric MRI (mpMRI). To enable a fair comparison between this method and other published models, and to enhance the reproducibility of results, experiments were conducted using the publicly available RSNA comprehensive dataset.^17^ To effectively build the network, reduce resource requirements, and improve clinical usability, the methodology was designed and executed using transfer learning, leveraging off‐the‐shelf deep features.^19^ To better represent the region of interest (ROI), 3D deep features from various mpMRI modalities were extracted from the output layer of pre‐trained natural video action recognition networks, as detailed in previous studies.^20^ To integrate features from different mpMRI modalities effectively, a Siamese neural network (SNN) was adopted as the backbone of the classifier.^21^ To further validate the reliability of the model, external validation was conducted using an open‐access dataset not included in the BraTS21 comprehensive dataset.

Major contributions of this study are:

(1) Single‐CPU execution: All model training and validation were performed on a single‐CPU platform, reducing resource requirements and enhancing clinical applicability.

(2) Externally validated TRIPOD 3a study: External validation was performed, and according to the Transparent Reporting for Individual Prognosis Or Diagnosis statement, this study is classified as a type 3a study.^22^

(3) Delta features boosted performance: The incorporation of delta deep features as input to the network, which enhanced the model's performance.

To facilitate transparency and reproducibility, we are making the source code of our study publicly available. The source code, alongside the deep features, data for statistical analysis, and supporting materials, can be accessed at: https://github.com/FORRESTHUACHEN/mpMRI_for_MGMT_Prediction‐

## 2 METHODS

**FIGURE 1:**
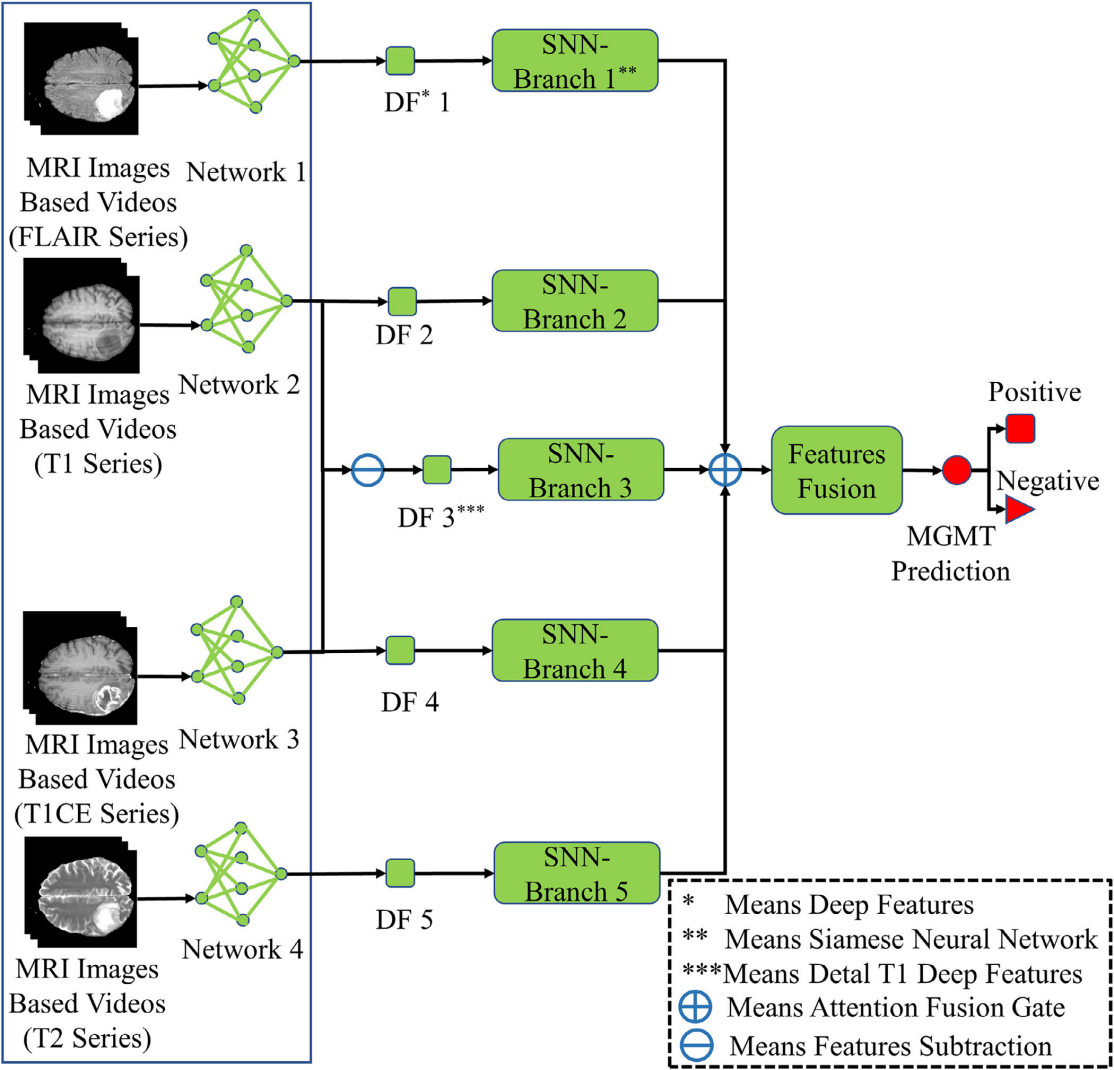
Pipeline of study.

Institutional review board approval was deemed unnecessary for this study due to the utilization of an open‐access data collection from BraTS21 competition^23^ and the cancer imaging archive (TCIA),^24^ where all patient‐specific private information had been anonymized in the MRI scans. The methodology of our study is delineated in Figure 1.

### 2.1 Patient cohorts

The primary dataset utilized in this study is the BraTS21 Challenge. This challenge includes two tasks: brain tumor segmentation and MGMT promoter methylation status prediction, with our focus on the latter. Multiparametric MRI scans of 1251 patients with glioblastoma were included in the challenge, of which 585 patients had publicly available MGMT status labels for the MGMT prediction task. The BraTS21 dataset comprises eight sub‐datasets, primarily collected from the TCIA platform, detailed descriptions of which are provided in Table S1. The BraTS21 dataset was employed for training and validating the MGMT prediction model, serving as the internal validation dataset.

For external validation, data were sourced from the University of California, San Francisco Preoperative Diffuse Glioma MRI (UCSF‐PDGM) dataset.^25^ This dataset includes mpMRI scans for 495 patients, with MGMT status available for 328 patients. Of these, 247 samples overlapped with the BraTS21 dataset, leaving 81 patients for external validation. A detailed index of patients eligible for external validation is provided in Table S2.

The MRI images included in this study exhibit variable slice thicknesses ranging from 0.43 to 6 mm and spatial resolutions ranging from 256*256 to 512*512. To ensure consistency, all images were preprocessed through the following pipeline: co‐registration to a common anatomical template using deformable registration, application of bias field correction per sequence, and intensity normalization across subjects. Temporal consistency within imaging sessions was maintained through motion correction, slice‐timing correction, and noise reduction. Finally, the image spacing for all images was normalized to 1.0 mm*1.0 mm*1.0 mm, and the image dimensions were standardized to 240*240*150 for subsequent analysis. Four imaging modalities were used to capture each patient's images: T1‐weighted pre‐contrast (T1), T1‐weighted post‐contrast (T1wCE), T2‐weighted (T2), and T2 fluid attenuated inversion recovery (T2‐FLAIR).

**FIGURE 2:**
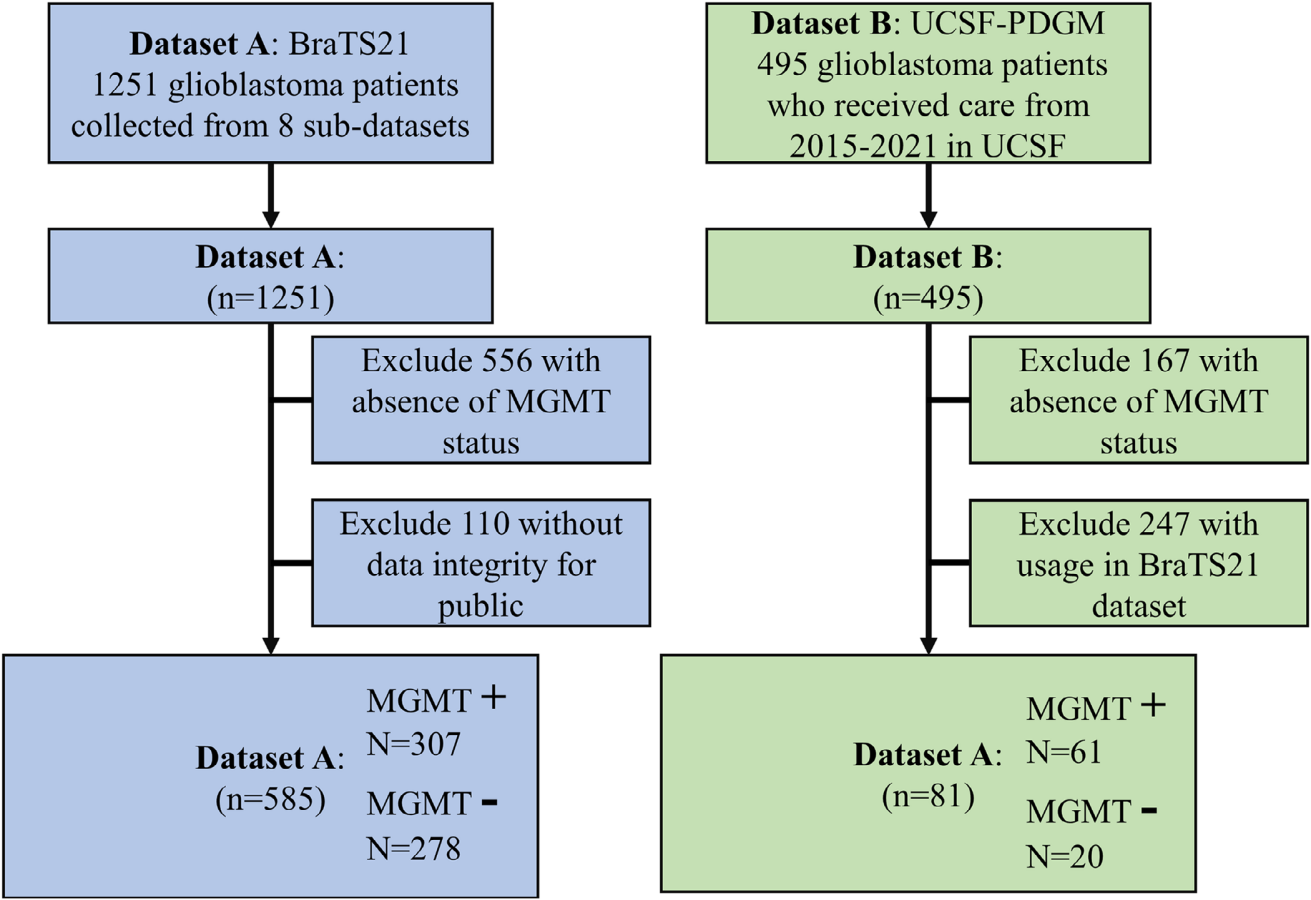
Flowchart shows samples exclusion for datasets.

The exclusion criteria for samples in both datasets are illustrated in the flowchart presented in Figure 2. Additionally, the statistical analysis of MGMT status for both datasets is also provided in Figure 2.

### 2.2 Off‐the‐shelf deep features extraction

The off‐the‐shelf deep features used in the subsequent analysis were extracted from the output layers of four networks‐ Inflated 3D ConvNet (I3D),^26^ SlowFast Networks,^27^ IRCSN Networks^28^ and video vision transformer (ViViT) networks.^29^ All networks were pre‐trained on the Kinetics‐400 dataset for the video action recognition task. The rationale for feature extraction in this manner will be discussed in the Discussion section. Feature extraction for the first three networks was conducted using the GluonCV deep learning platform^30^ while the pre‐trained ViViT model was downloaded from the Hugging Face platform. The specific pre‐trained models used for the first three networks were i3d_nl10_resnet101_v1_kinetics400, slowfast_4x16_resnet50_kinetics400, and r2plus1d_v2_resnet152_kinetics400, respectively. The detailed pre‐trained ViViT model used for feature extraction was ViViT‐L/16x2 FE.

To extract deep features from mpMRI images using our method, we converted mpMRI images to videos, with each slice representing a frame, using custom scripts. Images were normalized to the range 0–255 for the 1st–99th percentile of image intensities prior to conversion to videos. Tumor segmentation masks in the BraTS21 dataset were replaced with bounding boxes for feature extraction, size of bounding box was 128*128 in each frame, left boundary of the whole tumor segmentation across all frames and upper boundary of tumor was assigned as the upper left corner. Bounding box was applied all frames, it means there are 150 frames in each video for feature extraction, whole tumor was hold by bounding box. Ultimately, 400 deep features were extracted from I3D. The original meanings of each deep feature are included in Table S3.

In this study, the predictors consisted of deep features extracted from multiparametric MRI (mpMRI) data. Each sample was represented by a feature vector of size 5 × 400. The outcome variable was the MGMT promoter methylation status, a binary clinical endpoint where a value of 0 indicated an unmethylated MGMT promoter and 1 indicated a methylated MGMT promoter.

### 2.3 Classifier building

The pipeline of the classifier used in this study is illustrated in Figure 1, The backbone of the classifier is SNN, with detailed network architecture provided in Figure S1. Our model comprises five branches: four branches receive deep features from different imaging modalities, and the fifth branch receives the delta deep features of T1 and T1wCE modalities. Delta features were computed by performing image subtraction between the T1wCE and T1 modalities prior to deep feature extraction. The primary rationale for incorporating delta deep features is their demonstrated success in various imaging‐related clinical applications^31^.^32^ However, there are limited studies investigating the effectiveness of including delta features in network‐based classifiers. A self‐attention mechanism was employed to fuse features from different modalities and delta features.

### 2.4 Experiments and statistical analysis

The construction and validation of the classifier were carried out using Python 3.8, GluonCV 0.11, and Transformers 4.42.0 on a Core i5‐13600KF CPU with 32GB of RAM.^33^ During the internal validation phase, 585 samples from the BraTS21 dataset were selected, and performance was comprehensively tested using four repetitions of five‐fold cross‐validation. An early stopping strategy was employed during model training to prevent overfitting, with the network trained over 200 epochs. Performance metrics for both internal and external validations included recall, accuracy, and AUC.

To demonstrate the superior performance of the proposed method, comparisons were made with published methods on the BraTS21 competition dataset. The included references were methods proposed by Baid et al.,^17^ Saeed et al.,^18^ Emchinov,^34^ Kim et al.,^35^ Yogananda et al,^36^ and Faghani et al.^37^ Some references, such as^38^,^39^ were excluded from the comparison due to methodological differences. In the comparison, feature selection was a crucial procedure. A 3‐layer lightweight multilayer perceptron (MLP) served as a baseline model for comparison. This MLP utilized deep features extracted from T1wCE images using a ViViT network as its input. The model contained two hidden layers with 100 and 32 neurons, respectively, and incorporated a dropout rate of 0.4 to mitigate overfitting. Since some published methods did not report recall and accuracy, AUC was used as the primary metric, similar to the procedures used during the BraTS21 competition.

The ablation studies in this research were categorized into four classes:

1. mpMRI image modalities bias: Identifying the most suitable MRI modalities for MGMT status prediction was essential. Experiments were conducted to determine the appropriate modalities for this task. To evaluate the robustness of the model to missing imaging modalities—an important consideration for real‐world clinical deployment—we conducted a modality dropout ablation study.
2. Deep feature extraction networks: Deep features were extracted from four different video action recognition networks. Comparative performance analysis of models based on different features was conducted to identify the best network for feature extraction.
3. Feature integration marginal effect: The study also investigated the marginal effects of integrating deep features from multiple networks. Feature reproducibility is crucial for feature fusion, and concordance correlation coefficients (CCC) were used to measure feature reproducibility^40^.^41^
4. Delta T1 deep features: As a significant contribution of this study, the ablation study on the introduction of delta T1 deep features was performed to ensure the integrity of the study.
  For external validation, 81 samples from the UCSF‐PDGM dataset were used. This phase followed the same procedural framework as the internal validation based on the BraTS21 dataset.

## 3 RESULTS

### 3.1 Results of method comparison

**FIGURE 3:**
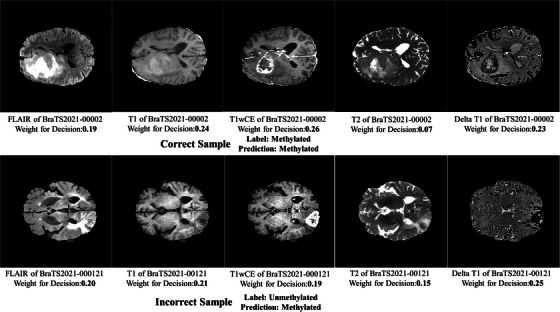
Two prediction examples with modality attention weights.

Wall‐clock times for training per fold and per‐case inference (including feature extraction from pre‐trained network) was 161.08 and 7.29 s based on listed CPU platform. Two prediction examples with modality attention weights for decision making were shown in Figure 3.

The performance of the established model for predicting MGMT status is presented in Table 1. As indicated in the table, the proposed method yielded an average AUC of 0.666 with a Standard Error of the Mean (SEM) of 0.031 when using deep features extracted from ViViT networks, achieving the highest AUC of 0.701 in a single run during the experiments. In contrast, the referenced methods achieved average AUCs ranging from 0.549 to 0.632 on the same dataset. Statistical analysis indicated that our proposed method significantly surpassed the performance of other methods (Wilcoxon rank‐sum test, same significance test hereafter).

**TABLE 1:** MGMT prediction results in BraTS21 dataset.

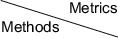	AUC**↑**	Precision**↑**	Recall**↑**
MLP	0.618 ± 0.039 p=0.01 ^*^	0.583 ± 0.032 p<0.01	0.575 ± 0.117 p<0.01
Baid et al.^17^	0.622^**^	–^***^	–
Saeed et al.^18^	0.631 ± 0.001 p=0.04	–	–
Emchinov^34^	0.596 ± 0.040 p<0.01	–	–
Kim et al.^35^	0.549 ± 0.054 p<0.01	0.616 ± 0.103 p<0.01	**0.687 ± 0.334**
Yogananda et al.^36^	0.580 ± 0.081 p<0.01	**0.660 ± 0.060**	–
Faghani et al.^37^	0.632 ± 0.027 p=0.07	0.630 ± 0.023 p=0.11	0.650 ± 0.160 p=0.19
Ours	**0.666 ± 0.027**	0.591 ± 0.021 p<0.01	0.630 ± 0.064 p<0.01

* means *p*‐value of Wilcoxon rank‐sum test compared with best method.

** reporting results without variance means it was not provided in original literatures.

*** means corresponding results were not reported in original literatures.

Moreover, the model achieved an average precision of 0.591 (SEM 0.021) and an average recall of 0.63 (SEM 0.064). As stated in the Materials and Methods section, AUC was adopted as the major metric for performance comparison. Therefore, based on the AUC results, it is evident that our method outperformed the compared methods.

**FIGURE 4:**
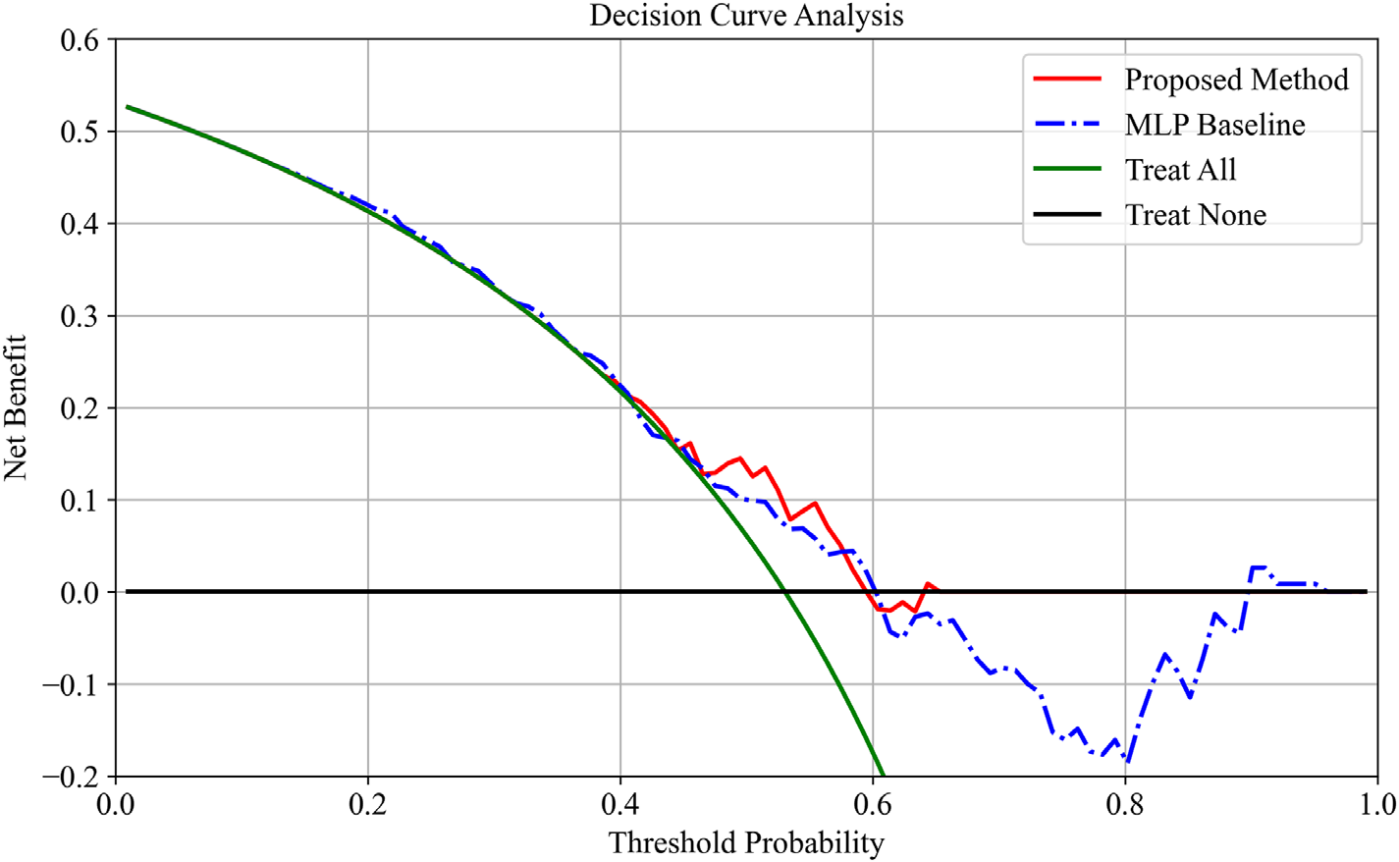
An example of decision curve analysis/net benefit figure of proposed method and MLP baseline.

As shown in Table 1, the MLP model achieved competitive performance compared to several existing methods, demonstrating the strong representational capacity of features derived from the ViViT network for medical image analysis. An example decision curve analysis (DCA) comparing the net benefit of the proposed method and the MLP baseline is presented in Figure 4.The results demonstrate that the proposed method achieves a superior net benefit, particularly when the threshold probability exceeds 0.6. In contrast, clinical decisions based on the MLP model introduce significant negative effects, especially in higher threshold ranges. This discrepancy may be attributed to the overconfidence and poor calibration often observed in pure neural network‐based classifiers, a phenomenon that has been extensively documented in prior research^42^.^43^

**FIGURE 5:**
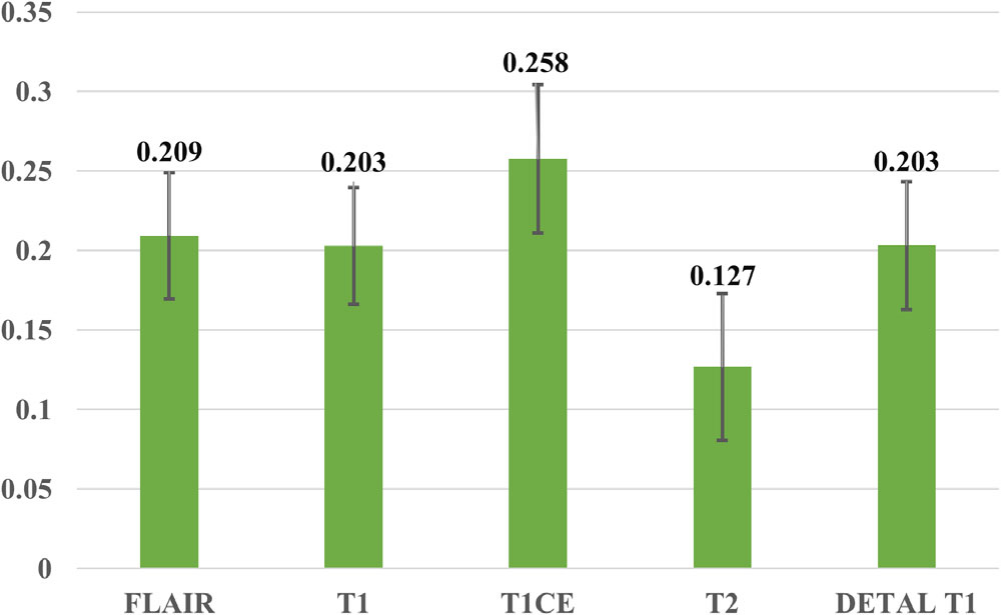
Average imaging modality weights: Internal validation performance (AUC = 0.65).

Modality‐level attention is important for real world deployment due to a possible phenomenon in multimodal analysis—the modality missingness^44^.^45^ Average imaging modality weights of an internal validation in BraTS 21 dataset was shown in Figure 5, trained model achieved an AUC of 0.65 in this validation. Results shown T1CE the most important modality for decision making and all modalities were essential for model and this result will be validated by another ablation studies.

Additionally, while high‐performance GPUs are essential for training the referenced methods, our model was constructed and validated using a single‐CPU platform, demonstrating that it is computationally efficient and can achieve superior results without the need for extensive computational resources.

### 3.2 Ablation study results of image modalities and feature extraction networks

Ablation study results on image modalities are shown in Table 2. The results indicate that there is an image modality bias for MGMT prediction based on mpMRI. All modalities were essential for the model, and the synergistic effect of combining multiple modalities significantly enhanced prediction performance. For single modality‐based prediction models, the post‐contrast T1‐weighted image model achieved relatively good performance. This finding aligns with clinical studies that have revealed the strong capability of post‐contrast T1‐weighted images to distinguish brain tumors from the background^46^.^47^

**TABLE 2:** Ablation study results on different MRI modalities and robustness of modality missingness.

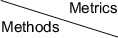	AUC**↑**	Precision**↑**	Recall↑
FLAIR^*^	0.6 ± 0.045 p=0.03	0.565 ± 0.051 p=0.44	0.677 ± 0.163 p=0.17
T1	0.614 ± 0.033 p=0.04	0.588 ± 0.035 p=0.83	**0.801 ± 0.031**
T2	0.612 ± 0.034 p=0.04	0.579 ± 0.047 p=0.66	0.698 ± 0.132 p=0.17
T1wCE	0.629 ± 0.051 p=0.26	**0.595 ± 0.051**	0.738 ± 0.078 p=0.17
Delta T1	0.569 ± 0.040 p<0.01	0.52 ± 0.044 p=0.06	0.729 ± 0.128 p=0.31
Modality missingness‐ FLAIR^**^	0.633 ± 0.032 p=0.25	0.592 ± 0.027 p=0.91	0.641 ± 0.098 p=0.01
Modality missingness‐ T1	0.631 ± 0.042 p=0.27	0.587 ± 0.034 p=0.52	0.622 ± 0.121 p=0.02
Modality missingness‐ T2	0.636 ± 0.018 p=0.19	0.572 ± 0.021 p=0.04	0.576 ± 0.144 p<0.01
Modality missingness‐ T1wCE	0.617 ± 0.037 p=0.10	0.594 ± 0.034 p=0.96	0.673 ± 0.089 p=0.17
Modality missingness‐ Delta T1	0.646 ± 0.017 p=0.52	0.59 ± 0.024 p=0.92	0.707 ± 0.159 p=0.18
All	**0.666 ± 0.027**	0.591 ± 0.021 p=0.90	0.630 ± 0.064 p<0.01

* Features extracted from pre‐trained ViViT networks;

** means FLAIR modality was excluded from classifier in modality missingness.

The results of this experiment are summarized in Table 2. A noticeable decrease in performance was observed under modality dropout conditions, reaffirming the importance of integrating multiple modalities. Furthermore, a strong negative correlation (*r* = –0.90, Pearson correlation coefficient) was found between the results of the modality dropout ablation and those from earlier experiments evaluating individual modality contributions, highlighting the complementary role of each modality in the overall model.

Regarding the ablation study on different pre‐trained feature extraction networks, the results are presented in Table 3. Statistical analysis showed that the choice of feature extraction networks significantly impacted the performance of the MGMT prediction model. Among the included networks, ViViT networks achieved the best performance. Moreover, the performance of the features in the MGMT prediction task had a strong correlation with the networks’ effectiveness in action recognition tasks ($r^{2}=0.93$, Pearson correlation coefficient). The performance of these networks in action recognition tasks can be found on some open‐access websites.^48^ Based on the results of the correlation analysis, the effectiveness of MGMT prediction models depends, to some degree, on the information representation ability of the deep features.

**TABLE 3:** Ablation study results on different pre‐trained feature extraction networks.

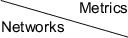	AUC**↑**	Precision**↑**	Recall**↑**
I3D network	0.6 ± 0.044 p=0.05	0.581 ± 0.045 p=0.69	0.66 ± 0.1 p<0.01
Slowfast network	0.572 ± 0.026 p<0.01	0.554 ± 0.045 p=0.18	0.627 ± 0.185 p<0.01
IRCSN networks	0.591 ± 0.024 p<0.01	0.563 ± 0.037 p=0.23	**0.76 ± 0.081**
ViViT networks	**0.666 ± 0.027**	**0.591 ± 0.021**	0.630 ± 0.064 p<0.01

### 3.3 Ablation study results of feature integrating marginal effect and delta T1 deep features

**FIGURE 6:**
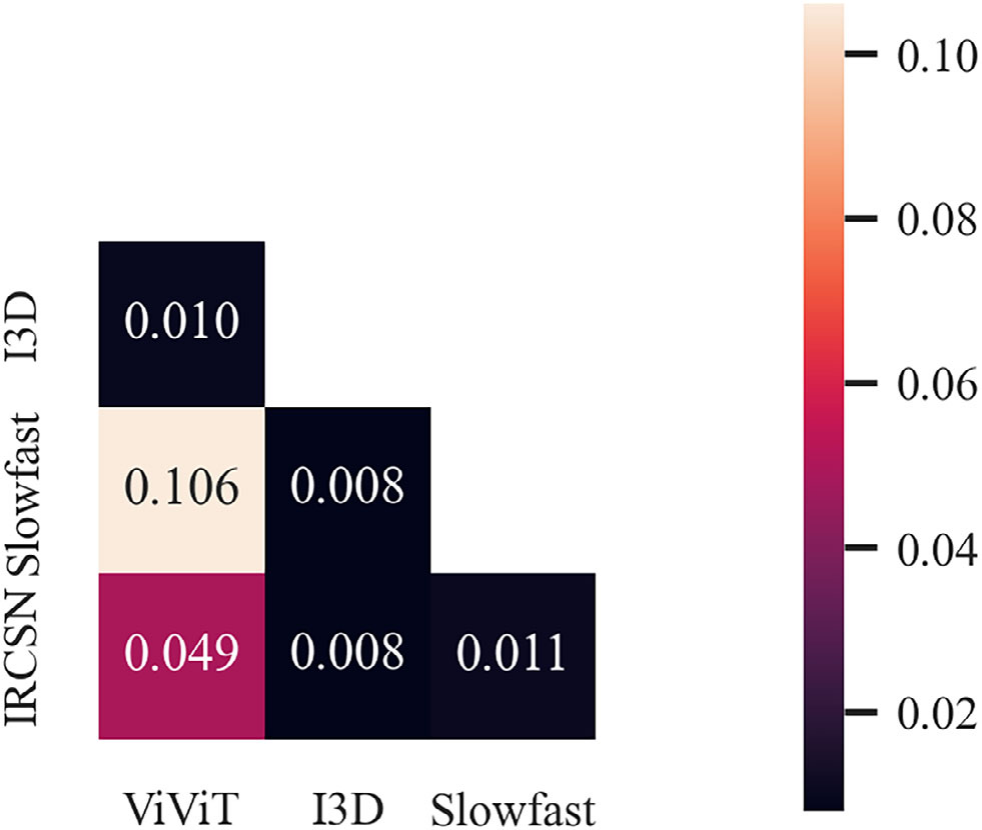
Average feature reproducibility results cross different networks.

Results of the ablation study for feature integration marginal effect, which involved features extracted from different networks, are shown in Table 4. The results demonstrated negative effects of integrating features for the MGMT prediction task. Possible reasons for this phenomenon include the high variance of the same features across different networks. Reproducibility analysis for each feature was executed, and the average feature reproducibility (CCC) across different networks is shown in Figure 6. The CCC values for each feature are detailed in Table S4. The results indicated low reproducibility of features across networks, with the average CCC value being lower than 0.15 in all analyses. However, the results of the feature integration marginal effect showed some correlation with the reproducibility of features ($r^{2}=0.58$). These experiments validated the proposed hypothesis of declining performance when integrating deep features from different networks. More details on this point are discussed in the Discussion section.

**TABLE 4:** Ablation study results of features integrating marginal effect from different networks.

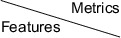	AUC**↑**	Precision**↑**	Recall↑
I3D + ViViT	0.597 ± 0.025 p=0.01	0.558 ± 0.015 p=0.03	0.513 ± 0.059 p=0.03
I3D + Slowfast	0.583 ± 0.026 p<0.01	0.563 ± 0.025 p=0.13	0.603 ± 0.149 p=0.75
I3D + IRCSN	0.602 ± 0.028 p=0.02	0.572 ± 0.013 p=0.16	0.526 ± 0.12 p=0.17
ViViT + Slowfast	0.612 ± 0.015 p=0.02	0.584 ± 0.015 p=0.84	0.573 ± 0.094 p=0.35
ViViT + IRCSN	0.61 ± 0.031 p=0.04	0.584 ± 0.043 p=0.61	0.585 ± 0.102 p=0.48
Slowfast + IRCSN	0.557 ± 0.031 p<0.01	0.551 ± 0.024 p=0.04	0.515 ± 0.063 p=0.03
I3D + ViViT + Slowfast	0.603 ± 0.027 p=0.02	0.570 ± 0.019 p=0.18	0.6 ± 0.084 p=0.59
I3D + ViViT + IRCSN	0.613 ± 0.023 p=0.03	0.581 ± 0.027 p=0.56	0.591 ± 0.040 p=0.33
I3D + Slowfast +IRCSN	0.587 ± 0.044 p=0.01	0.584 ± 0.035 p=0.74	0.626 ± 0.052 p=0.92
ViViT +Slowfast + IRCSN	0.611 ± 0.029 p=0.04	0.577 ± 0.044 p=0.58	0.563 ± 0.080 p=0.23
All features	0.585 ± 0.03 p<0.01	0.555 ± 0.023 p=0.05	0.589 ± 0.106 p=0.53
ViViT	**0.666 ± 0.027**	**0.591 ± 0.021**	**0.630 ± 0.064**

**FIGURE 7:**
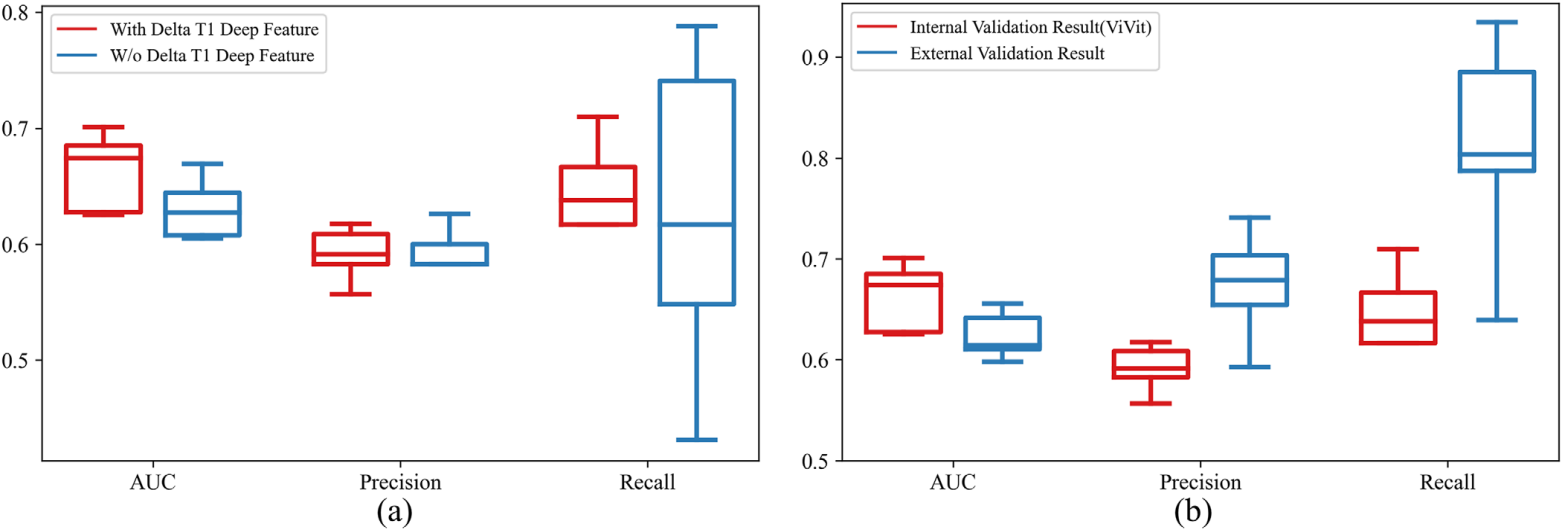
Results of (a) ablation study for introducing of delta T1 deep feature and (b) external validation.

Results of ablation study for introducing of delta T1 deep feature is shown in Figure 7a. Statistical analysis revealed a significant difference in the AUC metric (*P* = 0.03). No negative effects were found in other metrics by introducing the delta T1 deep feature. Therefore, we believe that introducing the delta T1 deep feature as a branch of input is a necessary procedure.

### 3.4 Results of external validation

External validation results are shown in Figure 7b, The proposed method achieved an average AUC of 0.624 (SEM 0.022), a precision of 0.674 (SEM 0.050), and a recall of 0.81 (SEM 0.101). When comparing these results to the internal validation results, no significant decrease in AUC was found (*P* = 0.08).

However, there was a significant increase in the metrics of precision and recall (*P* < 0.01). A possible reason for this increase could be the label distribution gap between the internal and external validation datasets. As shown in Figure 2, MGMT negative samples are minority samples (25%) in the external validation dataset. In contrast, MGMT negative samples are not a minority in the internal validation data.

## 4 DISCUSSION

MGMT is a critical prognostic factor in glioblastoma, and this study proposed a novel method for diagnosing MGMT status in a quick and non‐invasive manner based on mpMRI. To comprehensively utilize information from different mpMRI modalities, a SNN was adopted as the backbone of this network. Additionally, delta deep features of the T1 modality were integrated into the network to include more image information. Finally, external validation was conducted to increase the reliability of the study. Results demonstrated that our method achieved better performance compared to counterparts based on a single‐CPU platform.

In performance comparisons with previous studies, the proposed method achieved an average AUC of 0.666 over four repetitions of five‐fold cross‐validation, with the highest single‐run AUC reaching 0.701. These results surpass those reported in prior work. For instance, Saeed et al.^18^ reported an average AUC of 0.631 across a complete five‐fold cross‐validation, with a best single‐run result of 0.663, while Faghani et al.^37^ achieved an average AUC of 0.632 and a maximum of 0.660 under similar validation conditions. Furthermore, whereas most previous studies relied on high‐performance GPUs for model training—increasing computational barriers to deployment—our model was trained and executed on a CPU‐only platform, significantly reducing resource requirements. These results indicate that the proposed method not only improves predictive performance but also offers greater practical accessibility.

Deep features for transfer learning in medical image analysis are typically extracted via three primary methodologies: from the output or fully connected layer of a medical image analysis foundation model; from the bottleneck layer of domain‐specific encoder‐decoder networks (e.g., models tailored for CT or MRI); or from the output or fully connected layer of networks pre‐trained on natural image data. The rationale for extracting features from a natural video action recognition network, rather than from the bottleneck of a domain‐specific encoder‐decoder, is twofold. First, there is a lack of conclusive evidence demonstrating that features from encoder–decoder networks outperform those derived from models pre‐trained on natural images (e.g., on ImageNet or video data) within established multi‐class classification frameworks^49^.^50^ Second, the dimensionality of features from an encoder–decoder bottleneck is often prohibitively high (frequently around 10 000 features), whereas the output of a classification network provides a lower‐dimensional representation. This reduced feature space significantly streamlines model development and enhances the efficiency of both internal and external validation.^51^ A logical extension of this work is a feasibility study on using these 3D medical imaging foundation model features to predict MGMT promoter methylation status, a compelling direction for future research.

Medical image resampling is a common pre‐processing step in radiomics and deep feature extraction pipelines. However, as underscored by multiple studies^52^, ^53^, ^54^ this procedure often fails to eliminate the bias induced by variations in resolution and slice thickness. Feature harmonization methods, such as ComBat, are highly effective and often essential for removing these biases^55^. For instance, Kalantar et al. demonstrated that applying ComBat increased the mean percentage of robust features from 75.27% to 92.47%. Complementary to feature harmonization, medical imaging normalization^56^ have also shown considerable promise in mitigating batch effects. A significant challenge arises when acquisition parameters are unavailable, as is the case with the BraTS 2021 dataset, which lacks imaging protocol metadata. This absence renders most conventional feature harmonization and imaging normalization methods inapplicable. Consequently, subsequent research into unsupervised, generative model‐based approaches for multi‐center imaging normalization represents a critical and necessary direction. Techniques such as diffusion models^57^ and cycle‐consistent generative adversarial networks (CycleGAN)^58^ are particularly promising avenues for future investigation.

As illustrated in Figure 6, feature reproducibility across different branches of the SNN is low. The negative impact of integrating a heterogeneous deep feature branch on classifier performance can be attributed to three primary factors: representation misalignment, insufficient regularization, and optimization instability. First, when incorporating branches with highly heterogeneous features, each branch may learn representations specific to its unique initialization or training dynamics. Without explicit constraints—such as shared weights or feature alignment losses—these branches can diverge and capture disparate patterns, ultimately degrading classifier performance.^59^ Second, in the absence of regularization mechanisms that enforce similarity (e.g., contrastive learning, orthogonality constraints, or mutual information maximization), features from different branches may collapse into misaligned latent spaces, which in turn diminishes classification.^60^ Third, introducing additional branches expands the parameter space, exacerbating the instability of training dynamics. Gradient interference between branches can lead to inconsistent optimization trajectories, further impairing the classifier.^61^ In summary, high feature reproducibility across SNN branches ensures consistent embeddings, which improves within‐network similarity comparisons and enhances generalization. Reproducible features are inherently more robust to noise and initialization variations. Conversely, low reproducibility results in inconsistent embeddings, undermining pairwise similarity measures and classifier stability^62^.^63^

Limitations of this study include potential truncation errors arising from converting DICOM data to the input format for action recognition networks and possibly inappropriate window width and level settings due to imaging collection protocol biases. Additionally, the 3D deep features were not extracted using state‐of‐the‐art (SOTA) networks for action recognition, reflecting the exploratory nature of this study rather than an effort to find the optimal structure. Lastly, the interpretability of the proposed method remains a challenge, with conventional explainable artificial intelligence approaches proving ineffective, especially when incorporating high‐level features like deep features.

## 5 CONCLUSIONS

This study proposed a new method for diagnosing MGMT status in a quick and non‐invasive manner based on mpMRI. To comprehensively utilize information from different mpMRI modalities, a SNN was adopted as the backbone of this network. To streamline potential future clinical applications and reduce the complexity for stakeholders, the methodology was developed using off‐the‐shelf deep features. Additionally, delta deep features of the T1 modality were integrated into the network to include more detailed image information. To increase the reliability of the study, external validation was conducted. The results demonstrated that our method achieved better performance compared to counterparts based on a single‐CPU platform. Ablation studies showed the effectiveness of introducing delta T1 deep features, and external validation confirmed the reliability of the results across different datasets.

## AUTHOR CONTRIBUTIONS

**Junhua Chen**: Writing—review & editing; writing—original draft; visualization; validation; software; methodology; investigation. **Zhanghong Wang**: Writing—review & editing; writing—original draft; methodology. **Banghua Yang**: Writing—review & editing; writing—original draft; supervision; resources; project administration; investigation; funding acquisition.

## CONFLICT OF INTEREST STATEMENT

The authors declare that they have no known competing financial interests or personal relationships that could have appeared to influence the work reported in this paper.

## ETHICAL APPROVAL STATEMENT

Institutional review board approval was deemed unnecessary for this study due to the utilization of an open‐access data collection from BraTS21 competition and The Cancer Imaging Archive (TCIA), where all patient‐specific private information had been anonymized in the MRI scans.

## Data Availability

Data and supporting materials of this study will be made available in our online repository: https://github.com/FORRESTHUACHEN/mpMRI_for_MGMT_Prediction‐
